# Toward Personalized Neuroscience: Evaluating Individual‐Level Information in Neural Mass Models

**DOI:** 10.1002/hbm.70413

**Published:** 2025-11-16

**Authors:** Carlotta B. C. Barkhau, Clemens Pellengahr, Zheng Wang, Lukas Fisch, Ramona Leenings, Nils R. Winter, Jan Ernsting, Maximilian Konowski, Dominik Grotegerd, Susanne Meinert, Julia M. Hubbert, Judith Krieger, Tiana Borgers, Kira Flinkenflügel, Elisabeth J. Leehr, Frederike Stein, Florian Thomas‐Odenthal, Paula Usemann, Lea Teutenberg, Igor Nenadic, Benjamin Straube, Nina Alexander, Andreas Jansen, Christian Porschen, Tilo Kircher, John D. Griffiths, Hamidreza Jamalabadi, Udo Dannlowski, Tim Hahn

**Affiliations:** ^1^ Institute for Translational Psychiatry University of Münster Münster Germany; ^2^ Institute for Biomagnetism and Biosignalanalysis University of Münster Münster Germany; ^3^ Department of Psychiatry and Institute of Medical Sciences University of Toronto Toronto Ontario Canada; ^4^ Institute for Translational Neuroscience University of Münster Münster Germany; ^5^ Department of Clinical Psychology and Psychotherapy, Georg Elias Müller Institute of Psychology Georg August University of Göttingen Göttingen Germany; ^6^ Department of Psychiatry and Psychotherapy University of Marburg Marburg Germany; ^7^ Department of Anaesthesiology, Intensive Care and Pain Medicine University Hospital Münster Münster Germany; ^8^ Krembil Centre for Neuroinformatics, Centre for Addiction and Mental Health Toronto Canada; ^9^ Faculty of Medicine University of British Columbia British Columbia Canada; ^10^ Center for Mind, Brain, and Behavior (CMBB) University of Marburg Marburg Germany; ^11^ Medical School and University Medical Center OWL, Protestant Hospital of the Bethel Foundation, Department of Psychiatry Bielefeld University Bielefeld Germany

## Abstract

Macroscale brain modeling using neural mass models (NMMs) offers a framework for simulating human whole‐brain dynamics. These models are pivotal for investigating the brain as a complex dynamic system, exploring phenomena like bifurcations, oscillatory patterns, and responses to stimuli. While connectome‐based NMMs allow for the creation of personalized NMMs, their utility in capturing individual‐specific neural characteristics remains underexplored, with current studies constrained by small sample sizes and computational inefficiencies. To address these limitations, we employed an algorithmically differentiable version of the reduced Wong Wang (RWW) model, enabling efficient optimization for large datasets. Applying this to resting‐state fMRI data from 1444 samples, we optimized models with varying parameter complexities (*n* = 4, 658, and 23,875), which were derived from creating biologically plausible model variants. The optimized models achieved 4%, 19%, and 56% variance explanation in empirical functional connectivity (FC), respectively. Subject identification accuracy, based on simulated FC patterns, improved from < 1% (*n* = 4) to almost 100% (*n* = 23,875). Despite this precision, individual‐level correlations between model parameters and attributes like age, gender, or intelligence quotient were small (effect sizes: $\eta_{\text{partial}}^{2}\leq 0.03$, standardized β≤0.234). Machine learning analyses confirmed that these parameters lack the granularity to encode personal traits effectively. These findings suggest that, while current implementations of the RWW NMM can robustly replicate resting‐state dynamics, the resulting parameters may lack the granularity required to map onto individual‐specific behavioral metrics. This highlights a critical alignment problem: neural patterns and behavioral constructs such as intelligence may not correspond in a one‐to‐one fashion but instead represent higher‐level abstractions. Bridging this gap will require the development of new tools capable of uncovering the underlying mapping manifolds, likely situated at the level of functional dynamics rather than isolated parameters. Future efforts should build on individual‐level mechanistic modeling by exploring more expressive model classes and integrating richer sources of data, such as multimodal imaging or task‐based paradigms, to better capture individual variability in both neural dynamics and behavioral traits. Such approaches may ultimately help to bridge the gap between model‐based neural similarity and clinically meaningful personalization.

## Graphical abstract

Leveraging a differentiable reduced Wong Wang model, we optimized individual whole‐brain models for a large cohort (*n* = 1444) using rs‐fMRI, achieving up to 56% variance explanation. However, we found that current neural mass models, despite replicating resting‐state dynamics, lack the granularity to effectively model individual‐specific neural signatures.

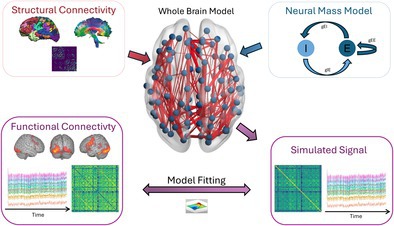

## 1 Introduction

Human brain dynamics are characterized by complex spatio‐temporal activity patterns that can be investigated using computational models. These models mathematically describe the evolution of brain activity over time and aim to simulate the temporal dynamics of neural activation. A commonly used approach is to describe large‐scale brain activity measurements, of the kind observed with noninvasive brain imaging techniques such as fMRI or EEG. This approach involves using anatomical connectivity matrices estimated from diffusion‐weighted MR tractography—yielding so‐called connectome‐based NMMs (Griffiths, Bastiaens, et al. 2022; Cabral and Griffiths 2024). To obtain individual‐level models—that is enabling the simulation of person‐specific dynamics—these models are informed by subject‐specific structural or functional brain data.

Recent studies have investigated the application of NMMs in different applications, for example to simulate epileptic seizures (Jirsa et al. 2017, 2023) or to distinguish between individuals with neurodegenerative diseases and healthy controls (HCs). Monteverdi et al. (2023) reported significant differences in NMM parameters between these groups using the reduced Wong Wang (RWW) model on a cohort of 33 patients. Similarly, Zimmermann et al. (2018) employed the same model on a larger dataset of 124 Alzheimer's patients and controls, but no significant group differences were observed; instead, they identified significant correlations between model parameters and cognitive performance scores. Iravani et al. (2021) found differences between HCs and ADHD patients in terms of attractor dynamics, using 447 HCs and 40 subjects with ADHD. More recently, Schirner et al. (2023) utilized a variant of the RWW model on the thus far largest sample of 650 participants, revealing associations between model parameters and intelligence quotient (IQ)‐related metrics.

Despite these findings, it remains challenging to establish an upper bound for the amount of individual‐level information captured by these models due to several limitations inherent to these studies. Firstly, many of these investigations are based on modest sample sizes (see table 4 in Ye et al. 2024), rendering them less suitable for generating broadly applicable conclusions. Small sample sizes pose a fundamental issue in scientific investigations as they reduce statistical power and increase the likelihood that results may occur by chance (Flint et al. 2021). This means smaller samples may not adequately capture the diversity and variability of the broader population, limiting the generalizability of the findings. Additionally, they increase the risk of biases and sensitivity to outliers, further compromising the reliability and replicability of study results.

Secondly, none of these studies examined whether model parameters covary with fundamental demographic variables such as age and gender. If the fitted parameter vectors do not recapitulate well‐established interindividual differences, this raises concerns about their sensitivity as individual descriptors and further reinforces the alignment gap articulated above: good neural fit does not guarantee behavioral alignment. We therefore use demographic effects as positive‐control checks for sensitivity.

Furthermore, the performance of these modeling approaches has yet to be benchmarked against empirical measures. If empirical fMRI demonstrates higher correlations with subject‐specific characteristics compared to the model parameters, then extensive optimizations may not be justified.

Lastly, much of the existing research has primarily concentrated on detecting differences between groups rather than capturing the variability between individuals. Incorporating machine learning (ML) techniques can address this gap by identifying complex, multivariate patterns that traditional statistical analyses might overlook. ML offers the potential to uncover subtle individual‐specific characteristics and enhance predictions by leveraging high‐dimensional data. Additionally, it can improve our understanding of interindividual variability, leading to more personalized insights and applications.

Against this backdrop, we now aim to quantify the upper bound of individual‐specific information captured by connectome‐based NMMs of resting‐state fMRI (rs‐fMRI), in a large study sample. To this end, we first adapted the algorithmically differentiable implementation (Griffiths, Wang, et al. 2022; Momi et al. 2023) of the two‐dimensional RWW model (Deco et al. 2014) to enable efficient deployment across large datasets. Second, we sought to verify that individuals can be identified from their personalized model parameter estimates at levels exceeding chance. Third, we evaluated the accuracy of the neural dynamics reconstructions by computing the explained variance between simulated and empirical functional connectivity (FC). Finally, we examined associations with a range of phenotypic and socio‐demographic variables (gender, age, body mass index [BMI], years of schooling [YoS], and IQ).

By implementing these enhancements, our approach offers several advantages for understanding individual‐specific neural dynamics. Adapting the model implementation to handle large samples enables the analysis of more diverse and representative data, improving the robustness and generalizability of our findings. Demonstrating that individuals can be differentiated beyond chance levels confirms the model's potential for meaningful individual‐level insights. Evaluating the accuracy of neural dynamics reconstructions ensures that the model achieves an optimal balance between abstraction and detail, which is essential for reliable interpretation. Lastly, measuring associations with phenotypic and socio‐demographic variables enriches our understanding by linking neural patterns with relevant individual characteristics, providing a more comprehensive view of how these factors interact with brain function.

## 2 Methods

### 2.1 Study Design and Participants

The data used in this work is part of the Marburg–Münster Affective Disorders Cohort Study (MACS) (Kircher et al. 2019; Vogelbacher et al. 2018). Data were collected at two sites (Marburg and Münster, Germany) using identical study protocols and harmonized scanner settings. The study was approved by the ethics committee of the medical faculties at the University of Marburg and the University of Münster in Germany. Participants provided written informed consent and received financial compensation for their participation. At the time of data analysis, measurements were available from 953 HC subjects, including second time point data for a subset of 491 subjects, resulting in a total of 1444 samples.

### 2.2 Procedures and Neuroimaging Data Modality

For preprocessing of the empirical diffusion‐weighted MRI tractography (DTI) and rs‐fMRI we used the publicly available and open‐source Connectivity Analysis Toolbox (CATO) (De Lange et al. 2023). We applied the Lausanne250 brain atlas (Cammoun et al. 2012) to both modalities. Due to data sparsity in subcortical regions, only the 219 cortical brain regions from the atlas were considered in this study.

Structural connectomes (SCs) were derived from DTI. The preprocessing procedure included the following steps: first, diffusion‐weighted images were preprocessed using FSL (Andersson and Skare 2002) and tracts were reconstructed using deterministic tractography. Then, we obtained a network of 219 cortical brain regions (Lausanne250 brain atlas) along with the reconstructed white matter streamlines between these brain areas for each participant. Network edges were reconstructed using deterministic streamline tractography based on the Fiber Assignment by Continuous Tracking algorithm (Mori and Van Zijl 2002). A single‐tensor reconstruction and deterministic tracking algorithm was chosen because it provides a reasonable balance between false negatives and false positives in fiber reconstructions (Sarwar et al. 2019). Edges between two nodes (i.e., brain areas) were included if at least three reconstructed streamlines connected them to balance the sensitivity and specificity of the resulting connectivity matrices (Zalesky et al. 2016; De Reus and Van Den Heuvel 2013). Each subject's network was finally stored in a connectivity matrix with rows and columns representing nodes and matrix entries representing edges (i.e., connectivity strength measured as the number of reconstructed streamlines). For further information about the DTI preprocessing, see Repple et al. (2023).

Functional connectomes (FCs) were derived from rs‐fMRI blood‐oxygenation‐level dependent (BOLD) time series. The time series underwent several preprocessing steps, including slice timing corrections using FSL sliceTimer and motion correction using FSL MCFLIRT (Jenkinson et al. 2002). Then the anatomical T1 image was used to parcellate the surface into brain regions with respect to the Lausanne250 brain atlas and compute the anatomical statistics of these regions. Additionally, motion metrics (framewise displacement [FD], change in signal intensity between frames [DVARS]) were computed for each frame following the implementation of Power et al. (2012).

The network reconstruction included the following steps: The covariates were removed from the signal intensity time series of the rs‐fMRI data, a band‐pass filter was applied to the rs‐fMRI data to remove noise frequencies, and frames that display significant motion artifacts were removed from the rs‐fMRI time series (maxFD: 0.25, maxDVARS: 1.5, minViolations: 2, backwardNeighbors: 1, forwardNeighbors: 0) (Power et al. 2012).

A band‐pass filter with cutoff frequencies of 0.01 and 0.1 Hz was applied, targeting the frequency range typically associated with resting‐state brain activity (Boubela et al. 2013). The filtering was performed using a zero‐phase Butterworth filter implemented in CATO (De Lange et al. 2023), effectively removing scanner drift and physiological artifacts such as respiration and cardiac signals without introducing phase distortions.

FC was then estimated between brain regions as the Pearson's correlation coefficient of the average intensity of these regions across the selected frames.

### 2.3 Brain Simulation Pipeline

Macroscale brain modeling using connectome‐based NMMs requires two key types of data: (1) measured physiological brain activity (e.g., time series of BOLD signals from rs‐fMRI or from EEG) and (2) an anatomical connectome derived from diffusion‐weighted MRI. The objective is to simulate the BOLD time series using the network structure provided by the anatomical connectome, complemented by an appropriately chosen NMM. In this study, we focus on modeling rs‐fMRI data using the SC as described above.

### 2.4 Neural Dynamics

To simulate neural activity across the cortex, we use the population activity at each ROI rather than the individual firing rates of single neurons. This approach allows us to simulate an entire brain model with a feasible computational load. To simulate signal processing at each ROI, we employed the RWW model as derived in Deco et al. (2013, 2014). In this model, each brain region consists of two neural masses: one representing the average behavior of an excitatory neural subpopulation and the other representing the behavior of an inhibitory subpopulation. This model was selected for its capacity to effectively represent the balance between excitation and inhibition (Deco et al. 2014), while also ensuring computational efficiency.

Each local population represents a node, and these nodes are interconnected through the subject‐specific SC to model the corresponding whole‐brain network dynamics. The activity at each node $i\in \left[1,\ldots ,N\right]$ is governed by a set of stochastic differential equations, which characterize the synaptic currents ($I_{i}^{E},I_{i}^{I}$), synaptic gating variables ($E_{i},I_{i}$), and firing rates ($R_{i}^{E},R_{i}^{I}$). The relationships are given by the following coupled nonlinear stochastic differential equations:

(1) $$\frac{d}{\mathrm{dt}}E_{i}=-\frac{1}{\tau^{E}}E_{i}+\left(1-E_{i}\right)\;\gamma R_{i}^{E}+\sigma v_{i}^{E}$$

(2) $$\frac{d}{\mathrm{dt}}I_{i}=-\frac{1}{\tau^{I}}I_{i}+R_{i}^{I}+\sigma v_{i}^{I}$$

where $\tau^{E}$ and $\tau^{I}$ are the decay times of excitatory and inhibitory synapses, respectively, γ is a kinetic parameter and $\sigma v_{i}^{E},\sigma v_{i}^{I}$ are uncorrelated Gaussian noise processes with mean 0 and standard deviation σ. The principal input to $E_{i}$ and $I_{i}$ are the population firing rates $R_{i}^{E}$ and $R_{i}^{I}$, which are expressed as functions of the input currents $I_{i}^{E}$ and $I_{i}^{I}$ as follows:

(3) $$R_{i}^{E}=\frac{a^{E}I_{i}^{E}-b^{E}}{1-e^{-d^{E}\left(a^{E}I_{o}^{E}-b^{E}\right)}},$$

(4) $$R_{i}^{I}=\frac{a^{I}I_{i}^{I}-b^{I}}{1-e^{-d^{I}\left(a^{I}I_{o}^{I}-b^{I}\right)}}$$

Parameters $a^{E},a^{I},b^{E},b^{I},d^{E},d^{I}$ govern the specifics of the conversion of input currents to population firing rates. The two input currents are computed as follow:

(5) $$I_{i}^{E}=\tanh\left(W^{E}I_{0}+w_{+}J_{\text{nmda}}E_{i}+GJ_{\text{nmda}}\sum_{j}L_{\mathrm{ij}}E_{j}-J_{i}I_{i}+I_{\mathrm{ext}}\right)$$

(6) $$I_{i}^{I}=\tanh\left(W^{I}I_{0}+J_{\text{nmda}}E_{i}+\mathrm{\lambda G}J_{\text{nmda}}\sum_{j}L_{\mathrm{ij}}E_{j}\right)$$

where $I_{\mathrm{ext}}$ encodes external stimulation, and is set to 0 when simulating resting state activity, as done in this study. $I_{0}$ denotes a steady external input, scaled by parameters $W^{E}$ and $W^{I}$ for the excitatory and inhibitory populations, respectively. $L_{\mathrm{ij}}$ represents the elements of the connectivity Laplacian, defined as L=D−C, where C is the (log‐transformed and unit‐normalized) tractography‐derived (adjacency) matrix that gives the connection strengths between network nodes i and j, and D is the diagonal matrix of node degree (i.e., the row sums of C). The term $\sum_{j}L_{\mathrm{ij}}E_{j}$ thus encodes the total summed input to node i from all other j nodes in the network. The parameter λ allows the removal of long‐range feedforward inhibition when set to 0 (Deco et al. 2014), which is what was done here. Parameters $J_{\text{nmda}}$ and $J_{i}$ represent the value of the excitatory synaptic coupling and the local feedback inhibitory synaptic coupling, respectively, while parameters $w_{+}$ and G scale the local and long‐range excitatory couplings, respectively.

In addition to deriving and introducing the two‐state RWW model, Deco et al. (2014) also introduced an iterative algorithm to keep the synaptic current terms within a specific biologically‐motivated range. Here we employ a different but related constraint as done in Griffiths, Wang, et al. (2022): we squash the input current variables in Equations (5) and (6) using a tanh function. This is a more mathematically well‐behaved way of specifying variable limits than explicit constraints or iterative algorithms.

In this paper we also adopt the following alternative notation from Deco et al. (2014) for several commonly discussed terms:

(7) $$g^{\mathrm{EI}}=J_{\text{nmda}}$$

(8) $$g^{\mathrm{IE}}=J_{i}$$

(9) $$g^{\mathrm{EE}}=w_{+}\times g^{\mathrm{EI}}$$

(10) $$g=G\times g^{\mathrm{EI}}$$

which define the within‐node excitatory‐to‐inhibitory ($g^{\mathrm{EI}}$) inhibitory‐to‐excitatory ($g^{\mathrm{IE}}$), and excitatory‐to‐excitatory ($g^{\mathrm{EE}}$) synaptic gains, as well as the long‐range global coupling g. In the following we will refer to these parameters as NMM parameters and fit them, to optimize the simulated rs‐fMRI.

To transform the state variables of the neural mass model (NMM) into a quantity that can be compared to the empirical rs‐fMRI signal, we use the Balloon–Windkessel model (Friston et al. 2000). This is common practice (see e.g., Deco et al. 2014) and captures much of the known biophysics of BOLD signal generation.

### 2.5 Parameter Optimization

The brain network model can be conceptualized as a differentiable model with customizable parameters, which influence how neural activity is processed at each node and transmitted to connected nodes. To achieve highly accurate simulations of brain activity, it is therefore essential to optimize these parameters.

In this study, we assessed three different versions of the RWW brain network model, each characterized by a different number of NMM parameters to be optimized. All other physiological and hemodynamic parameters were treated as fixed and are therefore not discussed here.

The first version, the globalized model (GM), is the commonly utilized four‐parameter RWW model. In this model, the varied parameters are g, $g^{\mathrm{EI}}$, $g^{\mathrm{IE}}$, and $g^{\mathrm{EE}}$.

The second version, the localized model (LM), still optimizes g globally, but allows $g^{\mathrm{EI}}$, $g^{\mathrm{IE}}$, and $g^{\mathrm{EE}}$ to vary at each individual node (brain region). This results in 658 optimized NMM parameters for the Lausanne250 atlas. To ensure that the parameters of different brain regions do not vary excessively, a small penalty term was added to the optimization's loss function.

The third version, the Connectivity Model (CM), optimizes g, $g^{\mathrm{EI}}$, $g^{\mathrm{IE}}$, and $g^{\mathrm{EE}}$ globally like the GM, but also allows for tuning of SC weights between brain regions. This approach leads to 23,875 optimized NMM parameters for the Lausanne250 atlas. After each optimizer step, the ${\mathrm{SC}}_{\mathrm{mod}}$ is clamped element‐wise to [0, 1]. To discourage large deviations from anatomy, we add $\lambda ∥{\mathrm{SC}}_{\mathrm{mod}}-{\mathrm{SC}}_{\mathrm{emp}}{∥}_{2},\left(\lambda =0.5\right),$ to the loss function. Symmetry and a zero diagonal are inherited from the upper‐triangular parametrization of the SC. Additionally, we quantified how closely the optimized structural connectivity remains anchored to the empirical SC by computing the edgewise Pearson correlation for each subject (summary statistics and distributions are provided in Section S1).

Similar to the approach of Griffiths, Wang, et al. (2022), we employed the ADAM algorithm (Kingma and Ba 2014) to optimize the NMM parameters. Compared to the model analyzed in Griffiths, Wang, et al. (2022), we implemented several adaptations. First, we introduced a learning rate scheduler to the ADAM optimizer, which resulted in more efficient optimizations. Second, we incorporated a “warm‐up phase” where we simulated 20 epochs of fMRI activity based on each individual's structural connectivity without modifying the NMM parameters. The output of this warm‐up phase served as the starting point for NMM optimization, allowing us to shorten the warm‐up phase in each subsequent optimization epoch and thereby reduce the overall optimization time.

**FIGURE 1:**
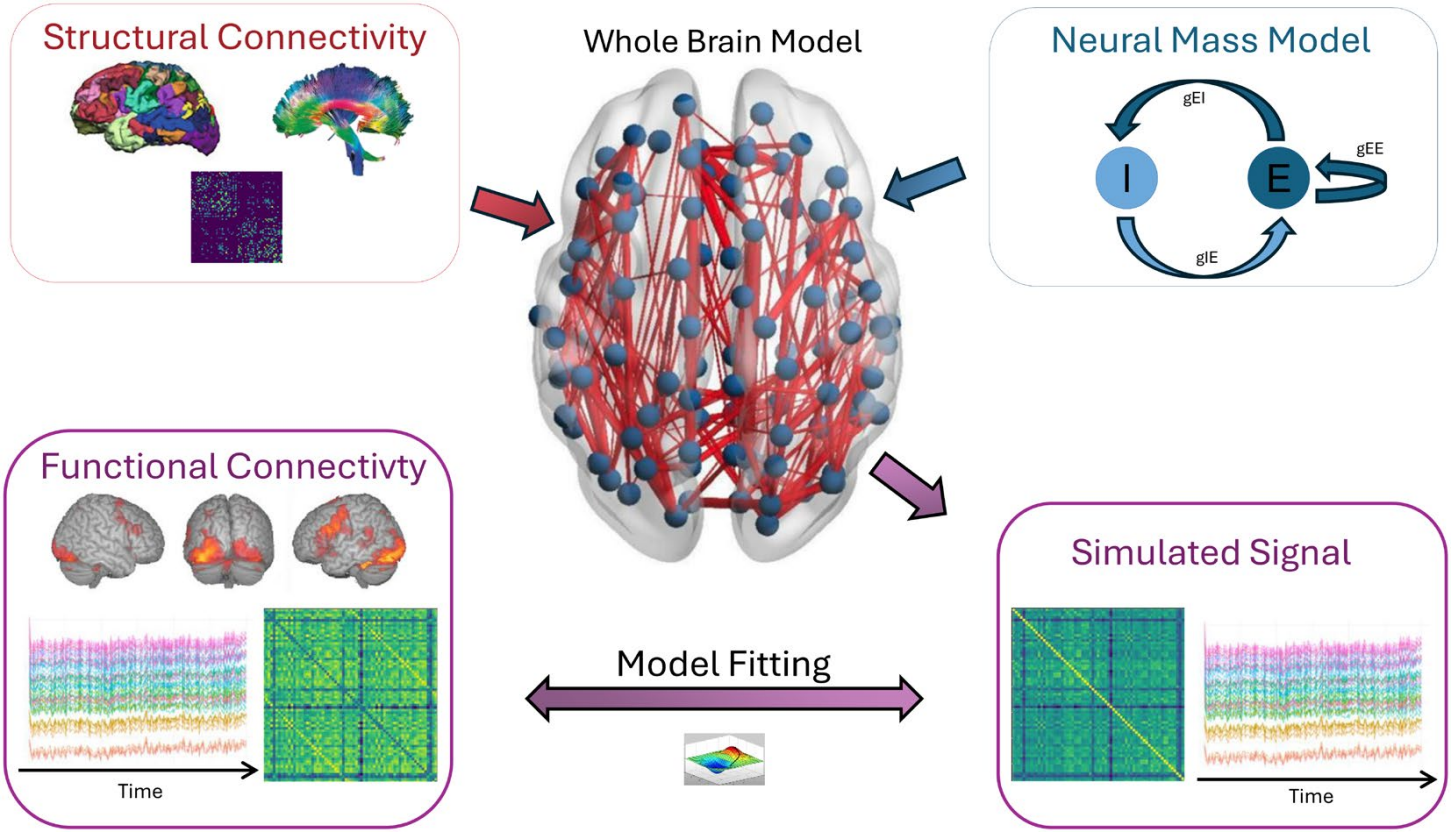
Schematic illustration of the whole brain, connectome‐based neural mass modeling framework. Input data comprise brain parcellations derived from T1‐weighted MRI and SC derived from diffusion‐weighted MRI, defining the connections between the model's nodes. An NMM specifies the dynamics of population‐level neural activity at each node (brain region). This integrated model enables simulation of fMRI time series, computation of a synthetic FC matrix, and comparison with an individual's empirical FC profile. Subsequent parameter optimization using the ADAM algorithm enhances alignment between simulated and empirical fMRI patterns.

The goodness of fit was evaluated by calculating FC matrices for both the simulated and empirical BOLD signals from their respective resting‐state fMRI time series. The Pearson correlation between the upper triangles (excluding the diagonal) of both FC matrices was computed. For the second and third models, we also included a penalty term in the goodness‐of‐fit computation. This approach aligns with common practices in the field, thus enabling appropriate comparisons of our results with existing literature. See Figure 1 for a schematic illustration of the whole brain model framework.

Using a differentiable version of the RWW brain network model and the ADAM optimizer, we were able to ensure computation times under 24 h per subject for 500 optimization epochs. Leveraging the high‐performance cluster, we could run multiple subjects in parallel.

To evaluate the degree of individualization present in our models, we attempted to identify the matching subject by comparing their simulated FC pattern to the full set of empirical FC patterns for the entire sample. Specifically, we calculated the Pearson correlation coefficient between each simulated FC matrix and every empirical FC matrix and assessed the likelihood that the correct empirical FC exhibited the highest correlation, ranked among the top three correlations, or fell within the top five.

In addition to evaluating the models, we computed the mean explained variance between the simulated and empirical FC for all three models to assess their performance. Explained variance was computed as the squared Pearson correlation coefficient (*R* ^2^) between the upper triangular elements of the empirical and model‐based FC matrices. To form a baseline for comparison, we calculated the mean explained variance over each participant's empirical SC and FC. Furthermore, we generated a mean FC matrix over all participants and correlated this matrix with each individual's FC. Additionally, for a subset of 491 participants with rs‐fMRI measurements from two distinct time points, separated by 2 years, we calculated explained variance between empirical measurements from the first and second time point.

We also characterized the isolated 2D node of the adapted Wong–Wang model via fixed‐point and stability analysis (full procedure and summary statistics are provided in Section S2) and re‐ran the optimization with a cohort‐mean SC to assess robustness against anatomical idiosyncrasy (full results are reported in Section S3).

### 2.6 Statistical Analysis of Model Parameters

The aim of this study is to evaluate whether the models' estimated NMM parameters possess sufficient depth to account for individual characteristics of the participants. Specifically, we examined gender, age, BMI, YoS, and IQ for each participant.

We employed an analysis of variance (ANOVA) model for gender and ordinary least squares (OLS) regression models for age, BMI, YoS, and IQ (continuous variables), predicting each target variable from the full set of model parameters. All models included age, gender, and scanning site as covariates of no interest (except that the outcome itself was not included as a covariate).

In the case of OLS, the general form of the regression was:

$$Y=\sum_{i=1}^{p}\beta_{i}X_{i}+\sum_{j=1}^{k}\gamma_{j}C_{j}+\epsilon$$

where Y is the continuous target variable (e.g., age), $X_{i}$ are the model parameters, $C_{j}$ are covariates (e.g., gender, site), and ϵ is the residual error.

For the ANOVA on gender, we modeled the categorical group variable using the same covariates (age, site) and calculated effect sizes using partial eta‐squared (*η* ^2^). All models were estimated independently for each parameter, consistent with standard univariate mass‐analysis procedures in neuroimaging. To account for multiple comparisons across parameters, we applied a false discovery rate (FDR) correction using the Benjamini–Yekutieli procedure with a threshold of q<0.05 (Benjamini and Yekutieli 2001).

Additionally, the values of the empirical FC matrix were utilized as inputs for the analysis, allowing a comparative analysis of the model parameters' performance against empirical rs‐fMRI.

For each modality, the variable showing the strongest effect was selected, and $\eta_{\text{partial}}^{2}$ (for categorical variables) or standardized β (for continuous variables) was calculated as a measure of effect size. Bootstrap confidence intervals were computed using the Bias‐corrected and accelerated (BCa) bootstrap method, including group stratification.

To quantify the predictive potential of the variables showing the largest group effect for the gender variable, a logistic regression was fitted on the confounded residuals of the linear models. Specifically, the covariates used in the analyses were regressed out of the data using the same linear models as described above, while excluding the group factor gender from the model first. The probabilities obtained from the logistic regression model were then used to plot a receiver operating characteristic (ROC) curve and calculate the area under the curve (AUROC).

To quantify the predictive potential of the variables showing the largest effects for continuous targets, we calculated mean absolute error (MAE), median absolute deviation (MAD), and both Spearman and Pearson correlation coefficients between the target variables and the predictions of a regression model using the variable showing the largest effect as a single predictor. The target variable showing the largest association with the predictor in this regression model was deconfounded with respect to the defined covariates, thereby estimating the individual effect on the target variable.

### 2.7 ML Analysis of Model Parameters

As ML algorithms are known for their ability to discover complex patterns across multiple parameters, we repeated the above tests using multivariate ML analysis instead of univariate statistical tests. To avoid data leakage, we only used the first time point for each subject, resulting in *n* = 953 samples. The input to the ML algorithms consisted of the models' optimized NMM parameters, and an algorithm was trained to predict each of the individual characteristics mentioned earlier. As a comparison, we also trained algorithms on the empirical rs‐fMRI. Therefore we flattened the upper triangular matrix of the empirical FC matrix and used these values as features.

Various ML models were trained, optimized and evaluated to predict the individual characteristics based on the model parameters. Each ML pipeline consisted of sequential preprocessing steps, including missing value imputation, feature normalization, and either univariate feature selection (based on ANOVA *F*‐values) or principal component analysis (PCA), followed by either a classification (for gender) or regression (for age, BMI, YoS, IQ) algorithm.

Imputation was done using the mean value of each feature within the training set (SimpleImputer, sklearn). Features were scaled using a RobustScaler to mitigate the influence of outliers. Feature selection was based on univariate F‐tests with thresholds at 5%, 10%, and 50% of retained features; alternatively, PCA was applied with full variance decomposition. The dimensionality reduction strategy was selected dynamically using a PHOTONAI Switch element during model optimization (Leenings et al. 2021).

The classifiers/regressors included support vector machines (SVM), random forests, logistic regression (with L1, L2, or elastic net penalties), *k*‐nearest neighbors (*k* = 5, 10, 15), Gaussian naïve Bayes and gradient boosting classifiers or regressors. Each algorithm underwent hyperparameter tuning within a nested cross‐validation framework (10 inner, 10 outer folds). For example, SVMs were evaluated with linear, polynomial, and RBF kernels across a wide range of *C*‐values (10^−8^ to 10^8^); logistic regression used *C*‐values from 0.0001 to 10,000.

Model performance was evaluated using *R* ^2^ scores for regression tasks and balanced accuracy for classification. All ML analyses were performed using PHOTONAI (Leenings et al. 2021), similar to the pipeline in Winter et al. (2024).

## 3 Results

Our differentiable version of the RWW model enabled us to investigate 1444 samples with three different levels of parameter granularity. The analysis revealed notable differences in the ability of each model to accurately recover the original subject's FC pattern within all subjects. The GM demonstrated limited success, with only 0.14% of cases correctly identifying the matching subject (i.e., achieving the highest correlation). When considering the top three and top five most correlated subjects, these rates increased marginally to 0.21% and 0.62%, respectively. In contrast, the LM performed better, exhibiting correct identification rates of 25.9%. This improved to 34.07% when examining the top three matches and 38.85% when evaluating the top five. The CM, however, does nearly always find the matching FC (99.79% top one). For comparison, attempting to match empirical SC to their respective FC patterns yielded substantially lower recovery rates, ranging from 0.07% (correct match) to 0.28% (top three) and 0.48% (top five)—and picking the right subject by chance has 0.07% top one, 0.21% top three, 0.34% top five probability. These results provide a useful baseline against which to assess the performance of our models.

**FIGURE 2:**
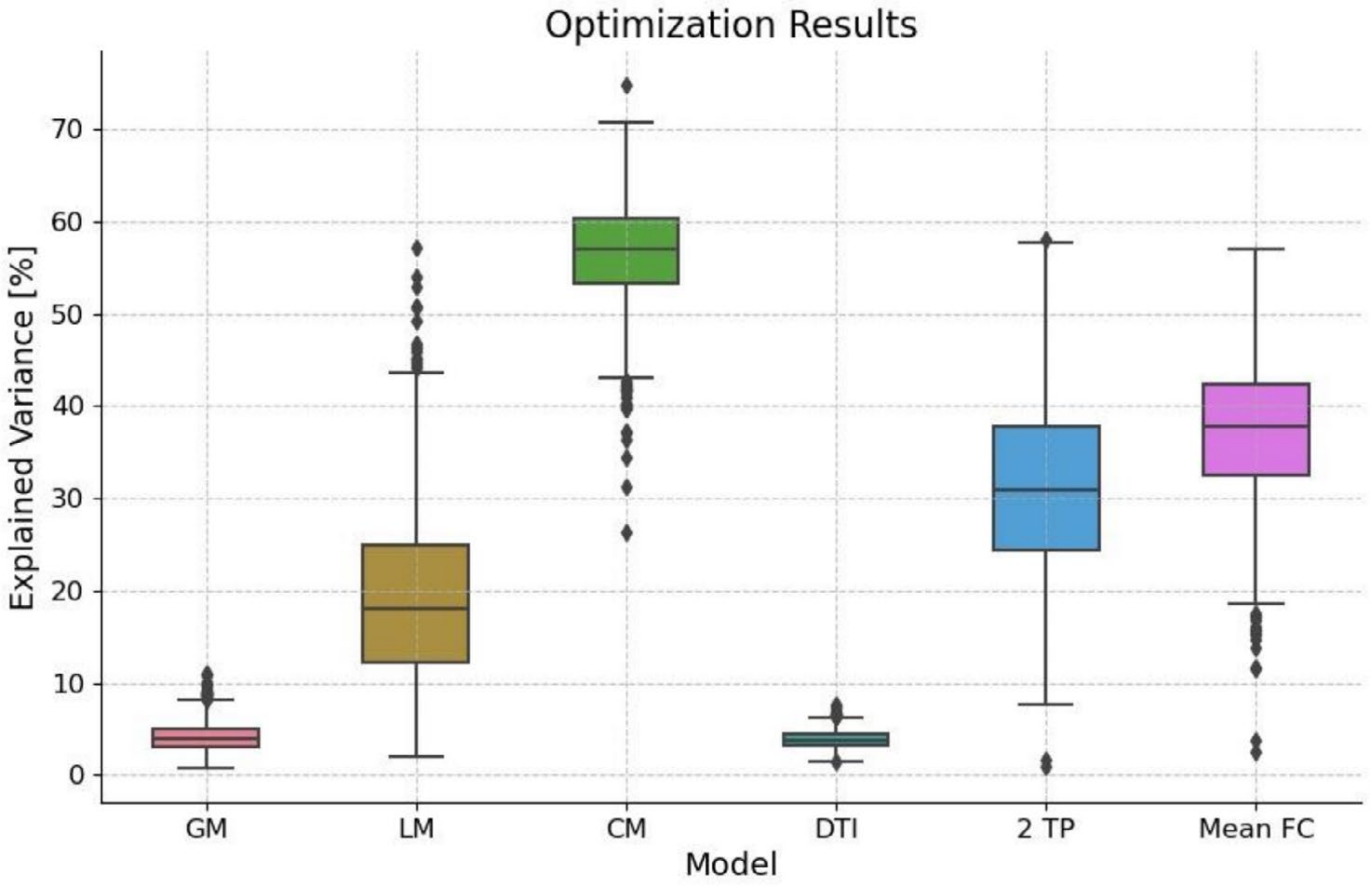
Optimization results: mean explained variance (%) of empirical rs‐fMRI data using simulated rs‐fMRI from different models (localized model [LM], globalized model [GM], connectivity model [CM]), empirical DTI, mean FC for *n* = 1444 samples, or empirical rs‐fMRI from a second time point (2TP) for *n* = 491 samples.

The optimization process yielded the following mean explained variances: for the GM, 4.12%; for the LM, 19.16%; and for the CM, 56.57%. As a baseline, the mean explained variance between each participant's empirical SC and FC was found to be 3.9%. When correlating individual FCs with the mean FC matrix across all participants, we observed an explained variance of 37.16%. Additionally, the variance explained by correlating empirical measurements from the two different time points, spaced 2 years apart, averaged 31.32%. Further details can be found in Figure 2.

In the next section, we will examine the results of the statistical tests. Across all modalities, the effect sizes for the variables displaying the largest differences between individual characteristics were small. The most substantial effects were observed for the CM in predicting age (standardized β=0.234) and gender $\left(\eta_{\text{partial}}^{2}=0.038\right),$ whereas the LM and GM demonstrated even less pronounced outcomes. Although some targets yielded statistically significant results (*p* < 0.05) for the models' parameters, the effect sizes remained small. Additionally, the empirical rs‐fMRI data consistently outperformed the models in terms of effect size, as evidenced in Table 1. It is important to highlight that mean absolute error (MAE) and standardized effect sizes (e.g., *β* coefficients) capture different aspects of model performance and are not necessarily aligned. While MAE reflects the average absolute deviation between predicted and true values in the original measurement units, standardized effect sizes quantify the strength of association relative to the variance in the data. As a result, it is possible for a model to achieve a lower MAE while exhibiting a smaller standardized effect size, particularly if the model produces predictions with reduced variance or shrinks toward the mean. This explains, for example, why the GM achieves the lowest MAE in predicting age, yet the empirical rs‐fMRI data shows a stronger standardized association with age.

**TABLE 1:** Results of statistical tests on parameters of the globalized model (GM), localized model (LM), connectivity model (CM) or empirical rs‐fMRI, employing ANOVA for gender and OLS for age, BMI, years of schooling, and IQ.

Characteristic	Model	*p*‐Uncorrected	*p*‐Corrected	$\eta_{\text{partial}}^{2}$	Overlap	BACC	AUROC
Gender	GM	0.001	0.012	0.007	89.6%	53.2%	0.54
	LM	< 0.001	< 0.001	0.026	85.8%	57.2%	0.59
	CM	< 0.001	< 0.001	0.038	83.3%	58.7%	0.61
	rs‐fMRI	< 0.001	< 0.001	0.057	79.6%	61.3%	0.65

Characteristic	Model	*p*‐Uncorrected	*p*‐Corrected	Standardized β	MAE
Age	GM	0.001	0.008	0.087	31.44
	LM	< 0.001	< 0.001	−0.145	34.68
	CM	< 0.001	< 0.001	0.234	35.6
	rs‐fMRI	< 0.001	< 0.001	−0.347	34.87
BMI	GM	0.075	0.63	−0.051	24.71
	LM	< 0.001	< 0.001	−0.127	21.93
	CM	< 0.001	< 0.001	0.168	24.33
	rs‐fMRI	< 0.001	< 0.001	0.219	24.09
Years of schooling	GM	0.065	0.542	−0.049	10.32
	LM	0.002	1	0.082	13.58
	CM	< 0.001	< 0.001	0.12	14.48
	rs‐fMRI	< 0.001	< 0.001	−0.12	14.33
IQ	GM	0.066	0.55	0.054	114.99
	LM	0.008	1	0.078	112.84
	CM	< 0.001	< 0.001	−0.119	115.41
	rs‐fMRI	< 0.001	< 0.001	−0.125	115.31

Across all models and ML algorithms, the best accuracies for predicting gender resulted in a balanced accuracy (BACC) of 0.74 for the CM. For age, the smallest mean squared error (MSE) was 110.17 (CM); for BMI, the MSE was 14.16 (CM); for YoS, the MSE was 7.1 (CM); and for IQ, the MSE was 163.4 (CM). The results for the best‐performing ML algorithm for each model and characteristic are presented in Table 2. However, it is noteworthy that the models' performances were outperformed by empirical rs‐fMRI, except for YoS and BMI, but even then, the explained variance of 0.05 and 0.16 for the CM is still small.

**TABLE 2:** Results of machine learning analysis using the parameters of the globalized model (GM), localized model (LM), connectivity model (CM) or empirical rs‐fMRI as features to predict individual characteristics.

Characteristic	Model	BACC
Gender	GM	0.53
	LM	0.65
	CM	0.74
	rs‐fMRI	0.87

Characteristic	Model	MSE	MAE	Explained variance
Age	GM	162.31	10.97	0.01
	LM	141.39	9.73	0.17
	CM	110.17	8.73	0.38
	rs‐fMRI	60.44	6.12	0.66
BMI	GM	16.09	3.14	0.01
	LM	14.86	2.98	0.09
	CM	14.16	2.94	0.16
	rs‐fMRI	14.86	2.98	0.09
Years of schooling	GM	7.48	2.38	0.02
	LM	7.35	2.4	0.02
	CM	7.1	2.34	0.05
	rs‐fMRI	7.14	2.22	0.03
IQ	GM	173.77	11.35	0.0083
	LM	172.54	11.16	0.003
	CM	163.4	10.9	0.06
	rs‐fMRI	151.81	10.33	0.2

## 4 Discussion

In this study, we introduced an effective method for optimizing NMM parameters. Utilizing a differentiable version of the RWW NMM enabled us to achieve precise optimizations within manageable computation times, thereby allowing us to optimize brain models for a substantial cohort of 1444 samples, far exceeding the sample sizes of previous studies. This innovation represents a crucial step toward obtaining reliable and reproducible results, addressing the concerns highlighted by Flint et al. (2021) regarding the risk of false positives associated with smaller sample sizes. Our efficient optimization process is essential for exploring sufficiently large datasets, ensuring that findings are both robust and representative. This contribution underscores the importance of scalable methodologies in enhancing the validity of neuroscientific research.

Beneath the commonly used GM, we additionally developed a new version of the RWW NMM—the LM. To our knowledge, this particular implementation of the LM has not been used previously. The LM variant of the brain model incorporates more degrees of freedom than the commonly used GM, resulting in higher correlations between simulated and empirical rs‐fMRI data while still maintaining a low‐dimensional representation compared to the empirical brain dynamics.

The third model, referred to as the CM, further increases the degrees of freedom in the optimization process, leading to even higher correlations with empirical data. However, this comes at the cost of dimensional simplicity; the WM no longer serves as a low‐dimensional representation, as it contains more parameters than the empirical rs‐fMRI connectivity matrix. Nonetheless it has been used in a comparable way, for example, in Schirner et al. (2023).

When trying to find the correct subject among all FCs, we observe that empirical DTI yields performances barely above chance levels. In contrast, the simulated FCs of all our proposed models surpass the predictive capabilities of empirical DTI. Consistent with expectations, the probability of accurate subject identification increases monotonically with the number of optimized parameters. Nonetheless, these outcomes offer preliminary evidence suggesting that the commonly employed GM struggles to incorporate meaningful individual‐specific information, as evidenced by its inability to retrieve the correct subject in more than 1% of instances. While the LM exhibits improved performance, its accuracy remains restricted, succeeding in approximately 25% of attempts. Conversely, the CM demonstrates exceptional proficiency, consistently identifying the correct subject, thereby potentially providing enhanced insight into individualized brain organization.

Examining the explained variance reveals similar trends across the models. While all models exceed the performance of empirical DTI, the GM does so only marginally. As anticipated, the explained variance increases with the number of optimized parameters, making the CM the top performer. Interestingly, the mean‐FC surpasses both the GM and LM, suggesting that these models' parameters might not contain substantial individual‐specific information. This observation provides an initial indication of the potential limitations in capturing unique subject characteristics.

Moreover, the statistical tests indicate that the NMM parameters lack the necessary depth to adequately account for individual characteristics. Although some tests yield results that are statistically significant (*p* < 0.05), the effect sizes are so small that these findings do not seem relevant for real‐world applications. Furthermore, empirical rs‐fMRI data also show statistically significant results with higher effect sizes compared to the NMM parameters. This indicates that there is no advantage in running complex optimizations and utilizing the resulting parameters in comparison to using the empirical rs‐fMRI data directly.

The same observation holds true for the ML analyses. While the NMM parameters lead to algorithms that perform above chance, the algorithms trained on empirical rs‐fMRI data mostly outperform those based on NMM parameters. Consequently, no advantage is gained from using the NMM parameters over the empirical rs‐fMRI data.

By examining a large cohort of 1444 samples, we were able to gain reliable insights into the ability of the models' parameters to represent individual characteristics. Our comprehensive analysis involved various methods, including reidentification tests, explained variance assessments, statistical evaluations, and ML approaches. The consistent trends observed across all these tests demonstrate the replicability of our findings, underscoring the importance of a large sample size in achieving robust and reliable results. Importantly, the scale of the dataset allowed us not only to improve modeling robustness but also to delineate the limits of the adapted RWW NMM with high confidence. While prior small‐*N* studies have reported parameter–behavior associations (Ye et al. 2024), our results indicate that such effects do not readily generalize under realistic noise and intersubject variability. In line with the alignment‐gap perspective above, good neural fit does not imply a one‐to‐one mapping to behavioral constructs; thus, large datasets are essential not only for detecting meaningful patterns but also for tempering—and, where appropriate, falsifying—overly optimistic assumptions about parameter granularity and behavioral relevance.

However, it is important to note that our study exclusively tested the RWW NMM, which is a suitable choice as it is often used in previous studies (e.g., Monteverdi et al. 2023; Zimmermann et al. 2018; Schirner et al. 2023) and it offers a good balance between complexity and feasible computation times. However, there remains the possibility that other NMMs could yield parameters with more individual‐specific information. Future research should explore a variety of NMMs to determine if different models can better capture individual characteristics.

Additionally, our analysis was based on rs‐fMRI sequences of 8 min. Investigating longer rs‐fMRI sequences could potentially enhance the robustness and reliability of both the NMM parameters and empirical data. Longer sequences might better capture the dynamic fluctuations in brain activity, thereby providing richer datasets for model optimization and analysis.

In summary, while our findings highlight the limitations of the RWW NMM and the rs‐fMRI sequence duration used in this study, they also point to promising future directions. Exploring alternative NMMs and incorporating longer fMRI sequences could provide new insights and potentially improve the utility of NMM parameters in capturing individual brain dynamics.

## Supporting information


**Figure S1:** Distribution of Pearson *r* between empirical and modified structural connectivity across subjects after applying the CM.
**Figure S2:** Local RWW regime composition by model (medians across subjects). Bars show the median fraction of nodes per regime for GM, LM, and CM; colors indicate monostable, bistable, and no stable FP.
**Figure S3:** Per‐subject local regime fractions. For each subject, we plot the fraction of nodes that are monostable, bistable, or no stable FP. GM and CM are summarized as median ± IQR whiskers, reflecting their tight concentration at ~100% monostable. LM is shown as boxplots with jittered subject points, revealing greater variability (median 95% monostable, 5% no stable).
**Figure S4:** Network stability margin per subject, with *m* > 0 stable, *m* = 0 critical and *m* < 0 linearly unstable for the connectivity model (CM).
**Figure S5:** Mean explained variance (%) of empirical rs‐fMRI data using simulated rs‐fMRI from different models (localized model [LM], globalized model [GM], connectivity model [CM]) with mean‐SC as input.

## Data Availability

The data that support the findings of this study are available on request from the corresponding author. The data are not publicly available due to privacy or ethical restrictions.

## References

[hbm70413-bib-0001] Andersson, J. L. R. , and S. Skare . 2002. “A Model‐Based Method for Retrospective Correction of Geometric Distortions in Diffusion‐Weighted EPI.” NeuroImage 16, no. 1: 177–199. 11969328 10.1006/nimg.2001.1039

[hbm70413-bib-0002] Benjamini, Y. , and D. Yekutieli . 2001. “The Control of the False Discovery Rate in Multiple Testing Under Dependency.” Annals of Statistics 29, no. 4: 1165–1188. 10.1214/aos/1013699998.full.

[hbm70413-bib-0003] Boubela, R. N. , K. Kalcher , W. Huf , C. Kronnerwetter , P. Filzmoser , and E. Moser . 2013. “Beyond Noise: Using Temporal ICA to Extract Meaningful Information From High‐Frequency fMRI Signal Fluctuations During Rest.” Frontiers in Human Neuroscience 7: 168. 10.3389/fnhum.2013.00168/abstract. 23641208 PMC3640215

[hbm70413-bib-0004] Cabral, J. , and J. D. Griffiths . 2024. “Dynamic Brain Network Models: How Interactions in the Structural Connectome Shape Brain Dynamics.” In Computational and Network Modeling of Neuroimaging Data, 209–228. Elsevier.

[hbm70413-bib-0005] Cammoun, L. , X. Gigandet , D. Meskaldji , et al. 2012. “Mapping the Human Connectome at Multiple Scales With Diffusion Spectrum MRI.” Journal of Neuroscience Methods 203, no. 2: 386–397. 22001222 10.1016/j.jneumeth.2011.09.031

[hbm70413-bib-0006] De Lange, S. C. , K. Helwegen , and M. P. Van Den Heuvel . 2023. “Structural and Functional Connectivity Reconstruction With CATO—A Connectivity Analysis TOolbox.” NeuroImage 273: 120108. 37059156 10.1016/j.neuroimage.2023.120108

[hbm70413-bib-0007] De Reus, M. A. , and M. P. Van Den Heuvel . 2013. “Estimating False Positives and Negatives in Brain Networks.” NeuroImage 70: 402–409. 23296185 10.1016/j.neuroimage.2012.12.066

[hbm70413-bib-0008] Deco, G. , A. Ponce‐Alvarez , P. Hagmann , G. L. Romani , D. Mantini , and M. Corbetta . 2014. “How Local Excitation‐Inhibition Ratio Impacts the Whole Brain Dynamics.” Journal of Neuroscience 34, no. 23: 7886–7898. 24899711 10.1523/JNEUROSCI.5068-13.2014 PMC4044249

[hbm70413-bib-0009] Deco, G. , A. Ponce‐Alvarez , D. Mantini , G. L. Romani , P. Hagmann , and M. Corbetta . 2013. “Resting‐State Functional Connectivity Emerges From Structurally and Dynamically Shaped Slow Linear Fluctuations.” Journal of Neuroscience 33, no. 27: 11239–11252. 23825427 10.1523/JNEUROSCI.1091-13.2013 PMC3718368

[hbm70413-bib-0010] Flint, C. , M. Cearns , N. Opel , et al. 2021. “Systematic Misestimation of Machine Learning Performance in Neuroimaging Studies of Depression.” Neuropsychopharmacology 46, no. 8: 1510–1517. 33958703 10.1038/s41386-021-01020-7 PMC8209109

[hbm70413-bib-0011] Friston, K. J. , A. Mechelli , R. Turner , and C. J. Price . 2000. “Nonlinear Responses in fMRI: The Balloon Model, Volterra Kernels, and Other Hemodynamics.” NeuroImage 12, no. 4: 466–477. 10988040 10.1006/nimg.2000.0630

[hbm70413-bib-0013] Griffiths, J. D. , S. P. Bastiaens , and N. Kaboodvand . 2022. “Whole‐Brain Modelling: Past, Present, and Future.” In Computational Modelling of the Brain, edited by M. Giugliano , M. Negrello , and D. Linaro , 313–355. Springer International Publishing. 10.1007/978-3-030-89439-9_13. 35471545

[hbm70413-bib-0012] Griffiths, J. D. , Z. Wang , S. H. Ather , et al. 2022. “Deep Learning‐Based Parameter Estimation for Neurophysiological Models of Neuroimaging Data.” Neuroscience. 10.1101/2022.05.19.492664.

[hbm70413-bib-0014] Iravani, B. , A. Arshamian , P. Fransson , and N. Kaboodvand . 2021. “Whole‐Brain Modelling of Resting State fMRI Differentiates ADHD Subtypes and Facilitates Stratified Neuro‐Stimulation Therapy.” NeuroImage 231: 117844. 33577937 10.1016/j.neuroimage.2021.117844

[hbm70413-bib-0015] Jenkinson, M. , P. Bannister , M. Brady , and S. Smith . 2002. “Improved Optimization for the Robust and Accurate Linear Registration and Motion Correction of Brain Images.” NeuroImage 17, no. 2: 825–841. 12377157 10.1016/s1053-8119(02)91132-8

[hbm70413-bib-0016] Jirsa, V. , H. Wang , P. Triebkorn , et al. 2023. “Personalised Virtual Brain Models in Epilepsy.” Lancet Neurology 22, no. 5: 443–454. 36972720 10.1016/S1474-4422(23)00008-X

[hbm70413-bib-0017] Jirsa, V. K. , T. Proix , D. Perdikis , et al. 2017. “The Virtual Epileptic Patient: Individualized Whole‐Brain Models of Epilepsy Spread.” NeuroImage 145: 377–388. 27477535 10.1016/j.neuroimage.2016.04.049

[hbm70413-bib-0018] Kingma, D. P. , and J. Ba . 2014. “Adam: A Method for Stochastic Optimization.” arXiv. https://arxiv.org/abs/1412.6980.

[hbm70413-bib-0019] Kircher, T. , M. Wöhr , I. Nenadic , et al. 2019. “Neurobiology of the Major Psychoses: A Translational Perspective on Brain Structure and Function—The FOR2107 Consortium.” European Archives of Psychiatry and Clinical Neuroscience 269, no. 8: 949–962. 30267149 10.1007/s00406-018-0943-x

[hbm70413-bib-0020] Leenings, R. , N. R. Winter , L. Plagwitz , et al. 2021. “PHOTONAI—A Python API for Rapid Machine Learning Model Development.” PLoS One 16, no. 7: e0254062. 34288935 10.1371/journal.pone.0254062 PMC8294542

[hbm70413-bib-0021] Momi, D. , Z. Wang , and J. D. Griffiths . 2023. “TMS‐Evoked Responses Are Driven by Recurrent Large‐Scale Network Dynamics.” eLifeeLife 12: e83232. 10.7554/eLife.83232 PMC10121222 37083491

[hbm70413-bib-0022] Monteverdi, A. , F. Palesi , M. Schirner , et al. 2023. “Virtual Brain Simulations Reveal Network‐Specific Parameters in Neurodegenerative Dementias.” Frontiers in Aging Neuroscience 15: 1204134. 37577354 10.3389/fnagi.2023.1204134 PMC10419271

[hbm70413-bib-0023] Mori, S. , and P. C. M. Van Zijl . 2002. “Fiber Tracking: Principles and Strategies—A Technical Review.” NMR in Biomedicine 15, no. 7–8: 468–480. 12489096 10.1002/nbm.781

[hbm70413-bib-0024] Power, J. D. , K. A. Barnes , A. Z. Snyder , B. L. Schlaggar , and S. E. Petersen . 2012. “Spurious but Systematic Correlations in Functional Connectivity MRI Networks Arise From Subject Motion.” NeuroImage 59, no. 3: 2142–2154. 22019881 10.1016/j.neuroimage.2011.10.018 PMC3254728

[hbm70413-bib-0025] Repple, J. , M. Gruber , M. Mauritz , et al. 2023. “Shared and Specific Patterns of Structural Brain Connectivity Across Affective and Psychotic Disorders.” Biological Psychiatry 93, no. 2: 178–186. 36114041 10.1016/j.biopsych.2022.05.031

[hbm70413-bib-0026] Sarwar, T. , K. Ramamohanarao , and A. Zalesky . 2019. “Mapping Connectomes With Diffusion MRI: Deterministic or Probabilistic Tractography?” Magnetic Resonance in Medicine 81, no. 2: 1368–1384. 30303550 10.1002/mrm.27471

[hbm70413-bib-0027] Schirner, M. , G. Deco , and P. Ritter . 2023. “Learning How Network Structure Shapes Decision‐Making for Bio‐Inspired Computing.” Nature Communications 14, no. 1: 2963. 10.1038/s41467-023-38626-y PMC10206104 37221168

[hbm70413-bib-0028] Vogelbacher, C. , T. W. D. Möbius , J. Sommer , et al. 2018. “The Marburg‐Münster Affective Disorders Cohort Study (MACS): A Quality Assurance Protocol for MR Neuroimaging Data.” NeuroImage 172: 450–460. 29410079 10.1016/j.neuroimage.2018.01.079

[hbm70413-bib-0029] Winter, N. R. , J. Blanke , R. Leenings , et al. 2024. “A Systematic Evaluation of Machine Learning‐Based Biomarkers for Major Depressive Disorder.” JAMA Psychiatry 81, no. 4: 386–395. 38198165 10.1001/jamapsychiatry.2023.5083 PMC10782379

[hbm70413-bib-0030] Ye, C. , Y. Zhang , C. Ran , and T. Ma . 2024. “Recent Progress in Brain Network Models for Medical Applications: A Review.” Health Data Science 4: 0157. 38979037 10.34133/hds.0157 PMC11227951

[hbm70413-bib-0031] Zalesky, A. , A. Fornito , L. Cocchi , L. L. Gollo , M. P. Van Den Heuvel , and M. Breakspear . 2016. “Connectome Sensitivity or Specificity: Which Is More Important?” NeuroImage 142: 407–420. 27364472 10.1016/j.neuroimage.2016.06.035

[hbm70413-bib-0032] Zimmermann, J. , A. Perry , M. Breakspear , et al. 2018. “Differentiation of Alzheimer's Disease Based on Local and Global Parameters in Personalized Virtual Brain Models.” NeuroImage: Clinical 19: 240–251. 30035018 10.1016/j.nicl.2018.04.017 PMC6051478

