# Supplementary material for: Toward Personalized Neuroscience: Evaluating Individual‐Level Information in Neural Mass Models
Source: Hum Brain Mapp. 2025 Nov 16;46(16):e70413. doi: 10.1002/hbm.70413 (PMC12620565; doi:10.1002/hbm.70413)
Supplement: Supplementary file 1 — Figure S1: Distribution of Pearson r between empirical and modified structural connectivity across subjects after applying the CM. Figure S2: Local RWW regime composition by model (medians across subjects). Bars show the median fraction of nodes per regime for GM, LM, and CM; colors indicate monostable, bistable, and no stable FP. Figure S3: Per‐subject local regime fractions. For each subject, we plot the fraction of nodes that are monostable, bistable, or no stable FP. GM and CM are summarized as median ± IQR whiskers, reflecting their tight concentration at ~100% monostable. LM is shown as boxplots with jittered subject points, revealing greater variability (median 95% monostable, 5% no stable). Figure S4: Network stability margin per subject, with m > 0 stable, m = 0 critical and m < 0 linearly unstable for the connectivity model (CM). Figure S5: Mean explained variance (%) of empirical rs‐fMRI data using simulated rs‐fMRI from different models (localized model [LM], globalized model [GM], connectivity model [CM]) with mean‐SC as input. [file HBM-46-e70413-s001.pdf]

Supplements

S1: CM change on structural connectivity

In the CM, we directly optimized a dense SC weight matrix SC_{mod} (initialized at the empirical SC SC_{emp}) together with the model parameters. After each optimizer step, entries of SC_{mod} were clamped element-wise to [0,1]. Deviations from the empirical anatomy were penalized via an L2 term $\lambda \parallel SC_{mod} - SC_{emp} \parallel_2, (\lambda = 0.5)$, so the procedure encourages SC_{mod} to stay close to SC_{emp} but does not impose hard constraints on support or symmetry beyond what is present in the initialization. There is no hard "no-new-edges" mask: edges that are zero in SC_{emp} are discouraged but not forbidden to take small positive values if the optimizer finds it beneficial under the penalty. Symmetry and a zero diagonal are inherited from the empirical initialization.

Extent of change. Empirically, the optimized SCs remain close to anatomy: the edgewise Pearson correlation between SC_{mod} and SC_{emp} across subjects was mean $r = 0.805$ with $SD = 0.0718$. Thus, although the CM could in principle move weight outside the empirical support (subject to the L2 penalty and $[0,1]$ bounds), in practice the fits exhibit modest reweighting around the empirical scaffold rather than wholesale rewiring.

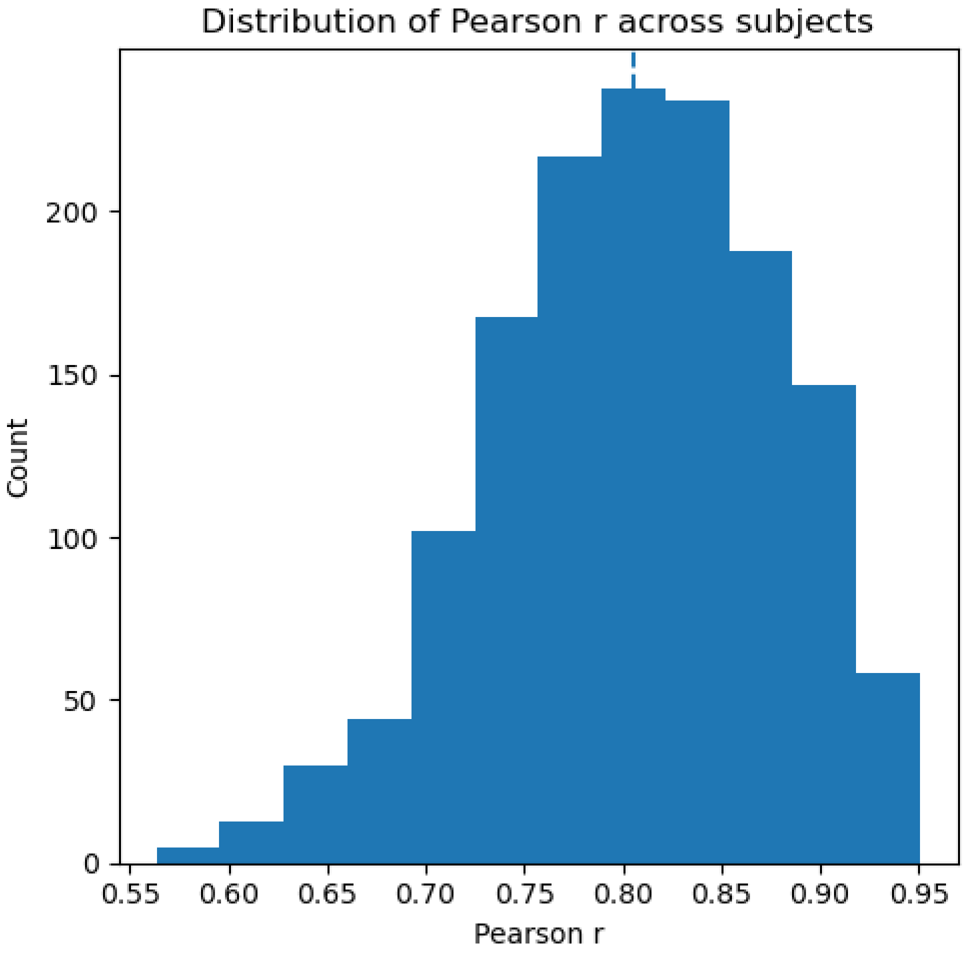

Figure S1: Distribution of Pearson r between empirical and modified structural connectivity across subjects after applying the CM.

S2: Model Dynamics

Local node dynamics

Methods. For each subject and each model (GM, LM, CM) we analyzed the isolated two-dimensional node (E, I) of the adapted RWW model, augmented with a Laplacian self-feedback term to capture the effect of structural coupling at the operating point. Concretely, in the E-equation we added $gk_i(\bar{E} - E_i)$, where g is the fitted global coupling (scaled as in training), k_i is the row-sum (degree) of the subject's full structural connectivity matrix, and $\bar{E}$ is the working-point mean field (held fixed during the local analysis). For the gains we used the subject's fitted g_{EE}, g_{EI}, g_{IE} (uniform for GM/CM; node-wise for LM when fitted), and the same transfer function and time constants as in the simulation model.

To classify regimes we scanned the state box $[0,1]^2$, refined candidate equilibria by a few Newton steps, and assessed linear stability from the Jacobian at each refined point (stable if

Re λ<0). For each node we counted the number of stable equilibria (mono- vs. bi-stability) and flagged nodes with no stable equilibrium in $[0,1]^2$ ("no stable FP"). We also checked for unstable foci (complex eigenvalues with positive real part) as oscillation candidates; these were essentially absent.

Results (medians across subjects). GM and CM are monostable-dominated: median fractions are 100% monostable, 0% bistable, 0% no-stable (IQRs are $[100, 100]$, $[0, 0]$, $[0, 0]$, respectively). LM shows a robust monostable majority but greater heterogeneity: median 95% monostable, 0% bistable, 5% no-stable with wide IQRs (monostable $[0.9, 97.3]$; no-stable $[2.7, 98.6]$). These summaries are visualized in the stacked-bar median composition (Supplementary Fig. S2) and the per-subject distribution plot (Supplementary Fig. S3), where GM/CM appear as median±IQR whiskers and LM as boxplots with subject-level jitter.

Interpretation. Across models, the adapted RWW node operates near stable fixed points, almost exclusively so for GM and CM, while LM permits a larger spread that includes a minority of nodes with no stable fixed point inside $[0,1]^2$. We find no evidence for intrinsic local oscillators (unstable foci are vanishingly rare), and local chaos is precluded by dimensionality (smooth 2-D ODE; Poincaré–Bendixson). Thus, rhythmic or complex patterns observed in full-network simulations should arise primarily from coupling and noise, not from self-sustained local limit cycles. Note that "no stable FP" means no stable equilibrium within the $[0,1]^2$ search box; equilibria just outside that box are not ruled out. Finally, the tanh input-squashing in our adaptation caps effective drive and shrinks oscillation/bistability-prone corners of parameter space, biasing fits toward fixed-point-dominated regimes without altering the core RWW structure.

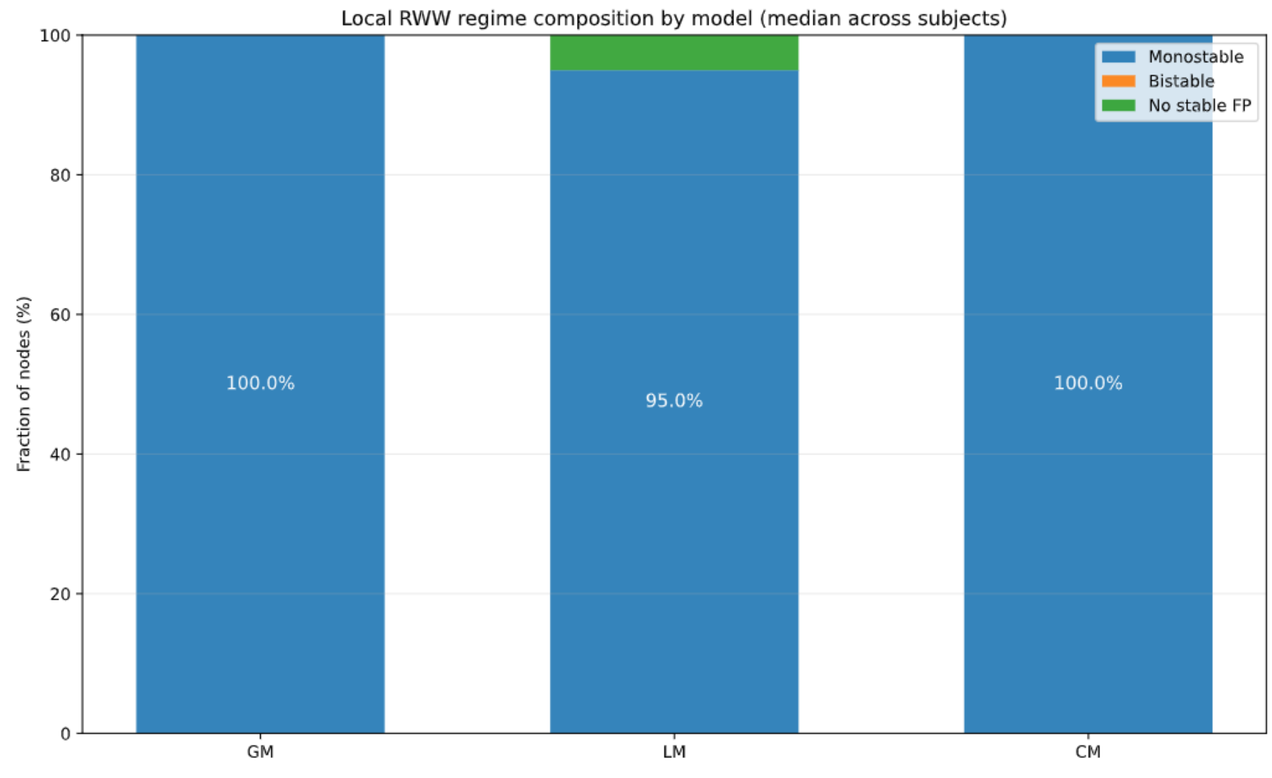

Figure S2: Local RWW regime composition by model (medians across subjects). Bars show the median fraction of nodes per regime for GM, LM, and CM; colors indicate Monostable, Bistable, and No stable FP.

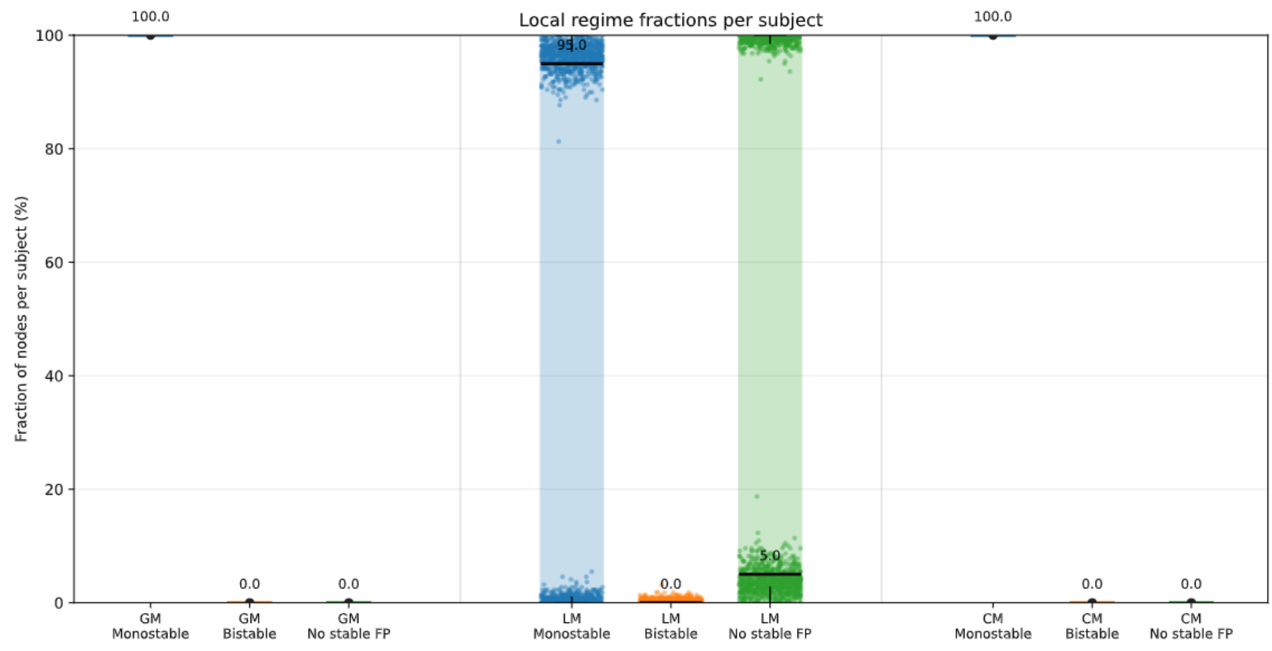

Figure S2: Per-subject local regime fractions. For each subject, we plot the fraction of nodes that are Monostable, Bistable, or No stable FP. GM and CM are summarized as median ± IQR whiskers, reflecting their tight concentration at ~100% monostable. LM is shown as boxplots with jittered subject points, revealing greater variability (median 95% monostable, 5% no-stable).

CM: Network-level linear stability

For the CM we also assessed the stability of the full coupled system. We linearized the

dynamics at the working point and defined the stability margin $m = -\max_i \mathrm{Re}\,\lambda_i(J)$, with J the Jacobian of the whole network (including structural coupling). Thus $m > 0$ indicates a linearly stable operating point, $m = 0$ is critical, and $m < 0$ is linearly unstable around that point. Across subjects, the margins cluster near zero (see Supplementary Fig. S4): 57.8% of subjects are stable ($m > 0$), with median $m = 0.008$ and IQR $[-0.033, 0.100]$. This places the optimized networks near the stability boundary (near-critical), consistent with the local-node results: nodes are fixed-point–dominated (monostable or bistable), and rhythmic/complex activity arises from coupling and noise, not from intrinsic local limit cycles or chaos.

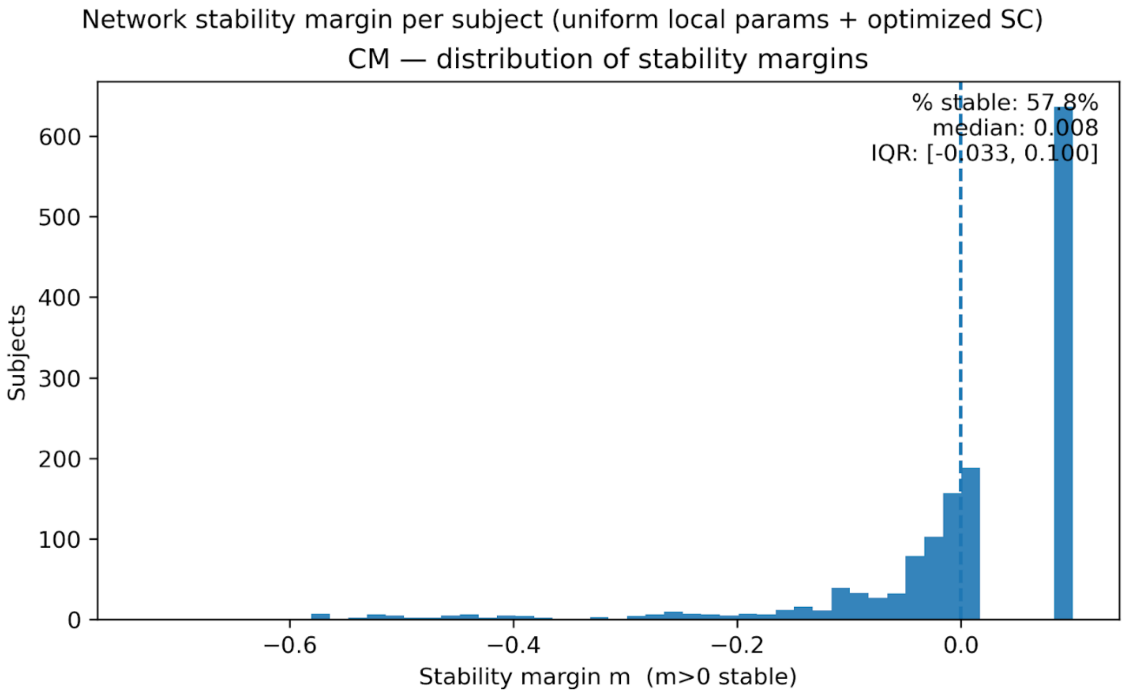

Figure S4: Network stability margin per subject, with m>0 stable, m=0 critical and m<0 linearly unstable for the connectivity model (CM).

Interpretation. Across models, the adapted RWW node operates near stable fixed points - predominantly monostable for GM/CM (with a subset bistable in the robust pass) and mixed mono/bi-stability for LM. Local oscillatory regimes are not supported (unstable foci are virtually absent), and local chaos is ruled out by dimensionality (smooth 2-D ODE; Poincaré–Bendixson). Thus, any rhythmic or complex patterns observed in network simulations arise from coupling and noise, not from intrinsic limit cycles or chaos of the isolated node. Mechanistically, our tanh squashing of inputs caps drive, shrinks oscillation-/bistability-prone corners of parameter space, and biases fits toward fixed-point-dominated regimes without altering the core RWW structure.

S3: Impact of individualized vs. cohort-mean structural connectivity (SC)

Setup. To test whether individual SC is necessary and whether a common SC could be more stable, we re-ran the full optimization using a single cohort-mean SC matrix (element-wise average of the individual SCs, same normalization and optimization protocol as in the main experiments). We then evaluated explained variance for FC correlation in the three models (GM, LM, CM).

Results. Using individualized SC, the models explained 4.12% (GM), 19.16% (LM), and 56.57% (CM) of the variance. When replacing individualized SC with a cohort-mean SC, the explained variance was 4.17% (GM), 22.84% (LM), and 54.96% (CM). Thus, the differences (mean-SC minus individualized) were +0.05 percentage points (pp) for GM, +3.68 pp for LM, and −1.61 pp for CM. See Supplementary Fig. S5.

Interpretation. Using a cohort-mean SC does not systematically outperform individualized SC. Performance differences are small (≤ ~4 percentage points on average): GM is essentially unchanged; LM shows a modest increase in average EV with mean SC; CM shows a modest decrease, indicating that subject-specific topology helps most when the model leverages the SC strongly (CM). While a common SC can be used as a practical baseline (and removes anatomical idiosyncrasies), it does not substitute for individualized anatomy when the goal is personalized FC modeling. We therefore retain individual SC in the main analyses and report the mean-SC results here for completeness.

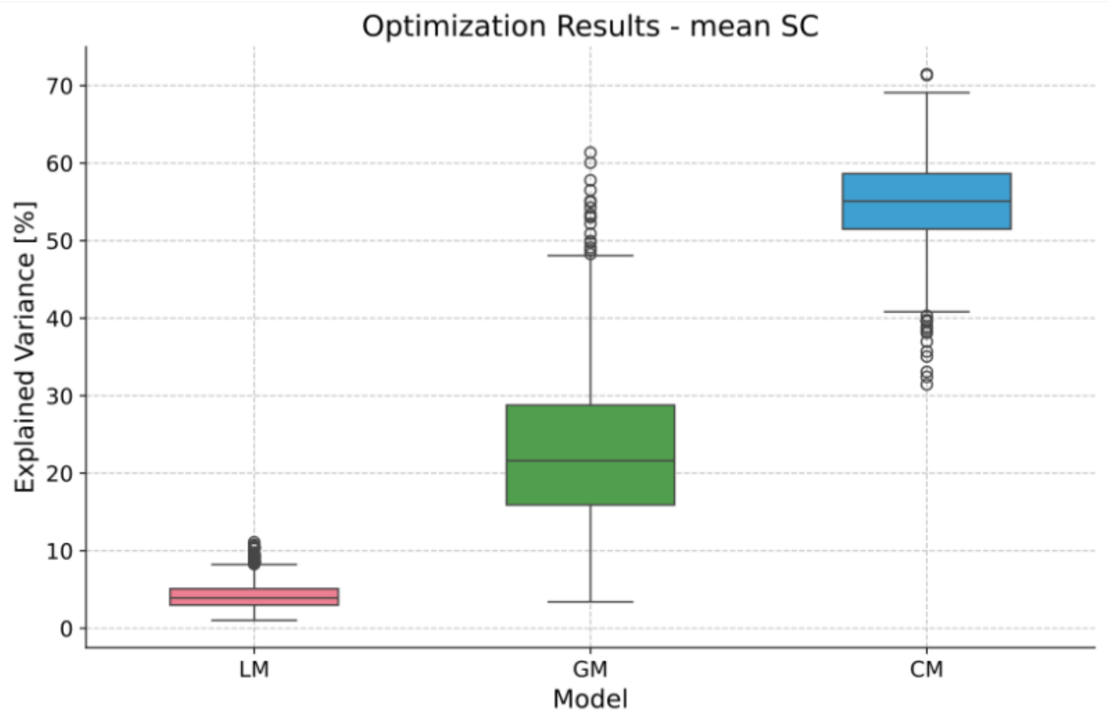

Figure S5: Mean Explained Variance (%) of empirical rs-fMRI Data using simulated rs-fMRI from different models (Localized Model [LM], Globalized Model [GM], Connectivity Model [CM]) with mean-SC as input.
